# Multiwall-carbon-nanotube/cellulose composite fibers with enhanced mechanical and electrical properties by cellulose grafting

**DOI:** 10.1039/c7ra11304h

**Published:** 2018-02-02

**Authors:** Shaobo Zhang, Feiran Zhang, Yanfei Pan, Liping Jin, Bo Liu, Yi Mao, Jintian Huang

**Affiliations:** College of Material Science and Art Design, Inner Mongolia Agricultural University Hohhot 010018 P. R. China jintian_h@163.com

## Abstract

Multiwall-carbon-nanotube (MWCNT)-cellulose/cellulose composite fibers with promoted mechanical and electronic activities were synthesized. Remarkably, the dispersion of MWCNTs in the composite fibers was facilitated through cellulose grafting, resulting in the tensile strength of the obtained MWCNT-cellulose/cellulose composite fibers being increased to 304.6 MPa with 10 wt% MWCNTs involved, which was almost 106.8% higher than that of pristine MWCNT/cellulose fibers with the same amount of MWCNTs. In addition, the electrical conductivity of the MWCNT-cellulose/cellulose composite fibers was enhanced to 1.3 × 10^−1^ S cm^−1^ with the dispersion of 10 wt% MWCNTs, which was almost 108 times higher than that of pristine MWCNT/cellulose fibers with the same amount of MWCNTs.

## Abstract 2

MWCNT-cellulose/cellulose composite fibers with enhanced mechanical and conducting properties were prepared *via* facilitating the dispersion of MWCNTs in fibers.

## Graphical abstract

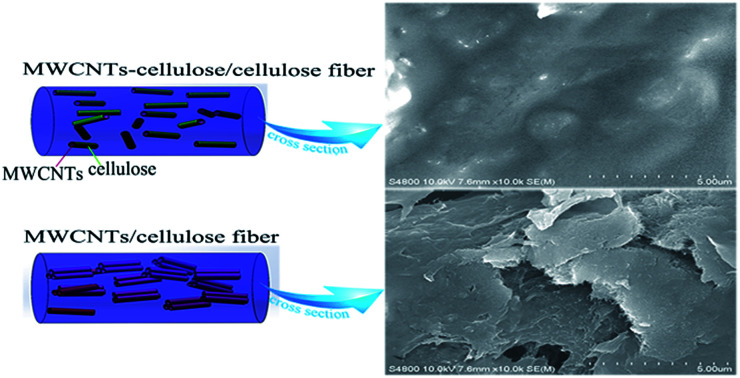

## 1. Introduction

Flexible electrical devices and wearable electrical appliances have been attracting significant attention due to their portability and integrability.^1^ As a major component of electronic devices, biocompatible and non-toxic conductive fibers have been attracting considerable attention in recent decades. For instance, they can be widely applied to wearable nano-generators,^2^ supercapacitors,^3^ batteries,^4^ actuators,^5^ and flexible solar cells.^6^ Cellulose, the most abundant polymer in nature, possesses a unique combination of renewable, biodegradable and biocompatible properties. Therefore, it is expected that cellulose would be considered as an inexhaustible resource to replace chemically derived compounds in some extremely desirable cases.^7^ However, there are some obstacles hindering the application of cellulose, such as difficult solubility owing to the large proportion of intra- and inter-molecular hydrogen bonds. Ionic liquids, a category of desirable green solvents, have been reported to be effective and promising solvents, with the merits of superior dissolving capacity, environmentally-friendly performance, recyclability, outstanding recoverability and variable structures.^8^ It was reported that natural cellulose can dissolve in ionic liquids effectively.^9^ Very recently, many polymers have been applied in the preparation of hetero-structural composites in ionic liquid systems, such as films, fibers, and foams.^10,11^ Carbon nanotubes (CNTs) have been paid considerable attention because of their exceptional mechanical, electronic, optical, and magnetic properties.^12–15^ These properties make CNTs excellent candidates to act as reinforcing agents in polymer composites. However, their application is hampered by the disadvantage of aggregation due to van der Waals interactions between the sidewalls of CNTs.^16^ Notably, the quality of the anisotropic nano-filler dispersion in the polymer matrix is directly correlated with its effectiveness for promoting mechanical and electrical functionality. Zhang synthesized regenerated-cellulose/multiwall-carbon-nanotube composite fibers in the ionic liquid 1-allyl-3-methylimidazolium chloride (AmimCl). Further exploring the mechanical and electrical activities of CNT/polymer composite fibers in ionic liquids and polymer matrix remains a challenge because of CNT aggregation.^11^ In previous reports, CNT/DNA fibers were prepared. The DNA facilitated the dispersion of the CNTs; the electrical conductivity of the fibers increased to 166.7 S cm^−1^, and the tensile strength was 101 MPa.^17^ Poulin reported dispersing CNTs in surfactant solution, recondensing the nanotubes in a flow of polymer solution to form a nanotube mesh. The electrical conductivity of the fibers was 10 S cm^−1^ at room temperature, and mean tensile strengths reached 150 MPa.^18^

**Fig. 1:**
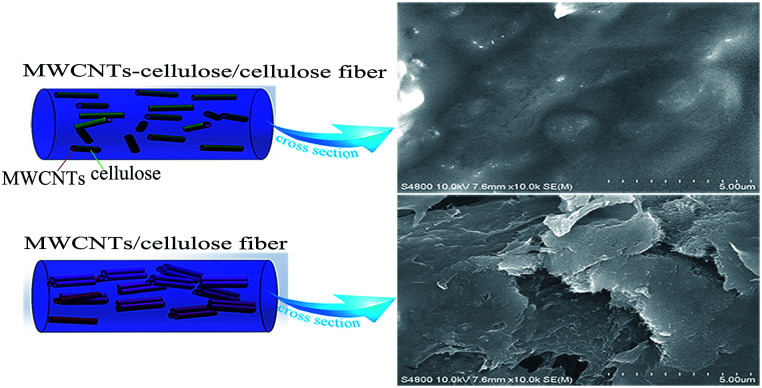
Schematic diagrams of MWCNTs in a MWCNT-cellulose/cellulose fiber and a MWCNT/cellulose fiber.

In our previous work, we developed natural polymer lignocelluloses, covalently grafting onto multiwall carbon nanotubes (MWCNTs) in the ionic liquid AmimCl, which facilitated the dispersion of MWCNTs in ionic liquids and lignocellulose films.^19^ Herein, MWCNT-cellulose/cellulose composite fibers with enhanced mechanical and conducting properties were prepared through cellulose being covalently grafted onto MWCNTs (Fig. 1).

## 2. Experimental

### 2.1 Materials

*Salix psammophila* cellulose powder was purchased from the Inner Mongolia Agricultural University, China. MWCNTs and carboxylated MWCNTs (MWCNT–COOH) were purchased from NanJing XFNANO Co, China. AmimCl was purchased from Lanzhou Yulu Fine Chemical Co. Ltd, China. Other reagents applied in this experiment were of analytical grade.

### 2.2 Preparation of MWCNT-cellulose^19^

MWCNT–COOH (0.7 g) and *N*,*N*-dimethylformamide (DMF, 1 ml) in SOCl_2_ (30 ml) is refluxed for 24 h; the residual SOCl_2_ is removed *via* distillation in a vacuum system. Subsequently, the obtained product is washed with acetone, filtrated and dried under vacuum. MWCNTs containing acyl chlorides (MWCNT–COCl) are obtained. Then, the above product (5 mg) is added into AmimCl (10 ml), sonicating for 30 min under vacuum. Cellulose (0.1 g) is mixed into AmimCl (10 ml), stirring for 1 h at 50 °C under nitrogen. These two solutions are mixed together, stirring for 24 h at 30 °C under nitrogen. The mixture is dispersed in distilled water, sonicated, and filtrated, respectively, then dried at 50 °C under vacuum. Finally, composites containing MWCNT-cellulose are obtained.

### 2.3 Preparation of MWCNT-cellulose/cellulose fibers and MWCNT/cellulose fibers

Selected amounts of MWCNT-cellulose are added to 8 ml of AmimCl to obtain 0 wt%, 2 wt%, 4 wt%, 6 wt%, 8 wt%, 10 wt%, 12 wt% and 14 wt% solutions, and the mixtures are ultrasonically stirred for 30 min at 100 °C under vacuum. 0.6 g of cellulose is added into the above solutions. The mixtures are stirred for 2 h at 100 °C under nitrogen. MWCNT-cellulose/cellulose composite fibers are spun from the solutions using dry-jet wet-spinning. The polymer dope is transferred to a vacuum oven with degasification. Then it is heated to 100 °C for 15 min, and extruded with nitrogen (0.5 MPa) through a single-hole spinneret (0.8 mm in diameter), entering a distilled-water coagulation bath, maintained at room temperature. Subsequently, the fibers are immersed in distilled water for 24 h and washed with distilled water at least five times to guarantee the removal of AmimCl. The fibers are dried for 48 h at 80 °C under vacuum; a pre-stressing force is applied at the ends of the fibers during drying in order to improve the alignment of the fibers. For comparison, MWCNT-cellulose is replaced with MWCNTs, with the same MWCNT content, and then MWCNT/cellulose fibers are obtained.

### 2.4 Characterization

X-ray photoelectron spectroscopy (XPS) was performed using a Thermo ESCALAB 250XI multifunctional imaging electron spectrometer, at 150 W, with an Al Kα source (*hν* = 1486.6 eV). Fourier transform infrared spectra (FTIR) of the samples were collected using a Nicolet Magna-IR 750 spectrometer with a wavenumber range of 4000–400 cm^−1^. X-ray diffraction (XRD) was performed using an XRD diffractometer (Omicron Nanotechnology, ESCA-14, Germany), which was operated at 40 kV/30 mA, with Cu Kα radiation at a wavelength of 0.154 nm. The Raman spectra were recorded with a Horiba LabRAM HR 800 spectroscope, using a 532 nm laser. Scanning electron microscopy (SEM) images of the fractured surfaces of the fibers were captured on a Hitachi S4800 microscope operating at 10 kV. The fibers were frozen in liquid nitrogen and immediately fractured. The fracture surface of the fibers was coated with a platinum layer before observation. Small-angle X-ray scattering (SAXS) measurements were obtained using a NanoSTAR U small-angle X-ray scattering system (Bruker, Germany), using a microfocus source with Cu-Kα radiation (45 kV, 0.65 mA). The mechanical properties of the MWCNT/cellulose and MWCNT-cellulose/cellulose composite fibers were measured using a universal testing machine (5940 Instron, USA) at a crosshead speed of 10 mm min^−1^. A gauge length of 20 mm was used. Mechanical tests were performed at room temperature. To ensure data accuracy and repeatability, at least five measurements were carried out for each composite fiber. The electrical conductivity of the fibers was measured at room temperature using a Solartron 7081 precision voltmeter with a four-probe method.

## 3. Results and discussion

### 3.1 Structure of MWCNT-cellulose

**Fig. 2:**
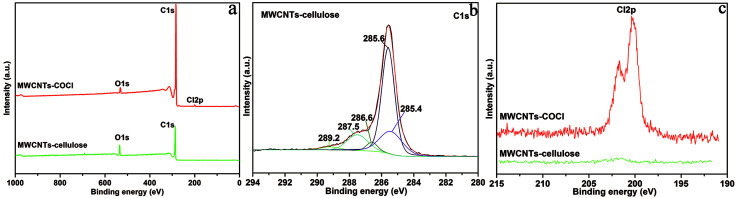
(a) XPS survey scan of MWCNT–COCl and MWCNT-cellulose. (b) XPS spectrum of the C 1s core level of MWCNT-cellulose. The spectrum curves were deconvoluted *via* Gaussian fitting. (c) Cl 2p narrow XPS scan of MWCNT–COCl and MWCNT-cellulose.

As shown in Fig. 2, the XPS survey spectrum demonstrated the following element content values: MWCNT–COCl had 98.45 at% carbon, 1.03 at% oxygen and 0.52 at% chlorine content; and MWCNT-cellulose had 90.13 at% carbon and 9.87 at% oxygen content. For further confirmation of the composite structure, the characteristic C 1s peaks of MWCNT-cellulose were deconvoluted *via* Gaussian fitting (Fig. 2b). The C 1s peak at 289.2 eV was ascribed to the ester carbon (O=C–O).^20^ The C 1s peaks at 287.5 eV and 286.6 eV arose from the bridging carbon (O–C–O) in the glucopyranose rings of cellulose and C–O in the ester groups and cellulose.^21,22^ The C 1s peaks at 285.6 eV and 285.4 eV represented the sp^2^ and sp^3^ hybridization of the C atom.^23^ In addition, the chlorine element of MWCNT–COCl and MWCNT-cellulose samples was investigated *via* XPS; the Cl 2p spectrum is exhibited in Fig. 1c. The Cl 2p peaks at 201.8 eV and 200.2 eV arose from the acyl chloride group (O=C–Cl) in MWCNT–COCl. However, there was almost no chlorine in MWCNT-cellulose, indicating that the acyl chloride group was involved in the reaction between MWCNT–COCl and cellulose. These XPS results indicated that cellulose was grafted onto the MWCNTs *via* an esterification reaction.

**Fig. 3:**
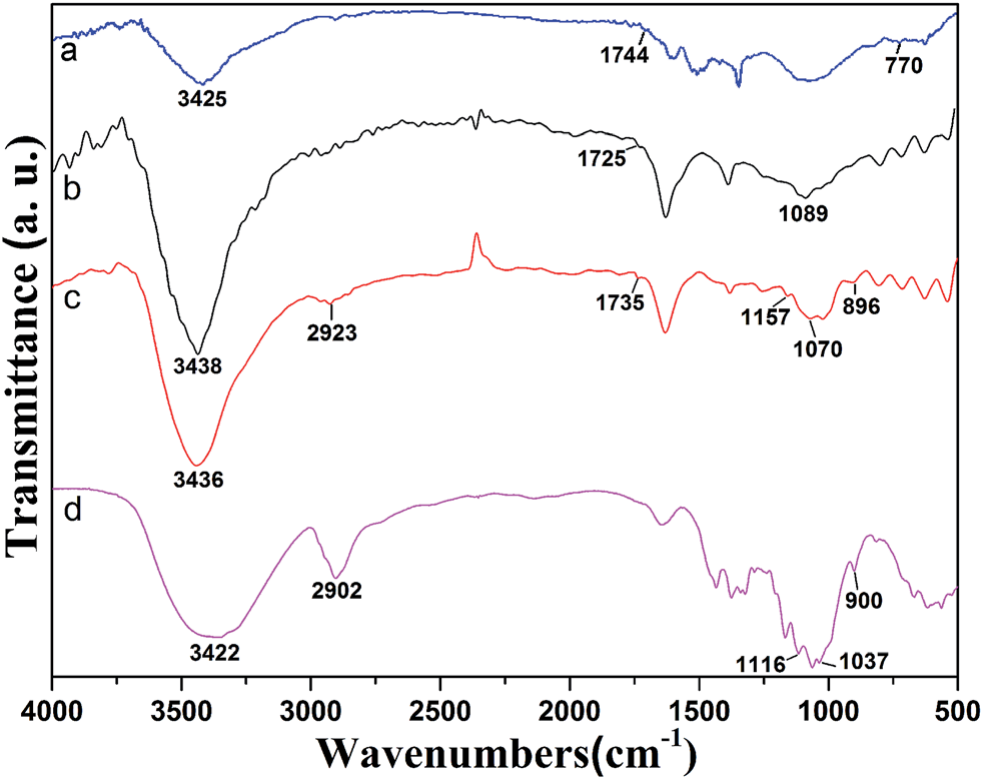
FTIR spectra of MWCNT–COCl (a), MWCNT–COOH (b), MWCNT-cellulose (c), and cellulose (d).

Fig. 3 exhibits FTIR spectra of MWCNT–COCl, MWCNT–COOH, MWCNT-cellulose and cellulose. The spectrum of cellulose shows a peak at 900 cm^−1^, which is indicative of beta-d-glucopyranosyl. Strong peaks at 1000–1200 cm^−1^ corresponded to the C–O stretching of the superposition of C–O–C groups, which was intra and between the anhydroglucose ring and the C–O of the secondary hydroxyl and primary oxhydryl. The pointed transmittances at 1116 cm^−1^ and 1037 cm^−1^ were ascribed to the C–O stretching of the secondary hydroxyl and primary oxhydryl, respectively. The peak at 2902 cm^−1^ was ascribed to C–H bond stretching. The broad peak at 3422 cm^−1^ was related to hydroxyl groups and hydrogen bonds. The spectrum of MWCNT–COOH (Fig. 3b) demonstrated that the peak at 1089 cm^−1^ could be ascribed to carboxyl C–O bond stretching. The transmittance at 1725 cm^−1^ was ascribed to carboxyl C=O bond stretching. The transmittance at 3438 cm^−1^ was related to O–H bond stretching. After acylation, as shown in Fig. 3a, in the FTIR spectrum of MWCNT–COCl the transmittance at 3438 cm^−1^ seen in MWCNT–COOH was weakened, and new transmittances at 1744 cm^−1^ and 770 cm^−1^ were ascribed to C=O and C–Cl in the acyl chloride group. After modification with cellulose, in the FTIR spectrum of MWCNT-cellulose (Fig. 3c), the characteristic bands from cellulose appeared at 2923 cm^−1^ and 896 cm^−1^, which were respectively ascribed to C–H bond stretching and beta-d-glucopyranosyl. Moreover, the transmittances at 1116 cm^−1^ and 1037 cm^−1^ from cellulose were absent after modification. This could be triggered by the secondary hydroxyl and primary oxhydryl in the reaction system. Additionally, in the FTIR spectrum of MWCNT-cellulose (Fig. 3c), the transmittance at 770 cm^−1^ from MWCNT–COCl was absent, caused by the C–Cl of the acyl chloride group being involved in the reaction. New transmittances from MWCNT-cellulose at 1735 cm^−1^, 1070 cm^−1^ and 1157 cm^−1^ were ascribed to C=O and C–O–C bond stretching of the ester. The transmittance results indicate the formation of covalent bonds between cellulose and MWCNTs.

**Fig. 4:**
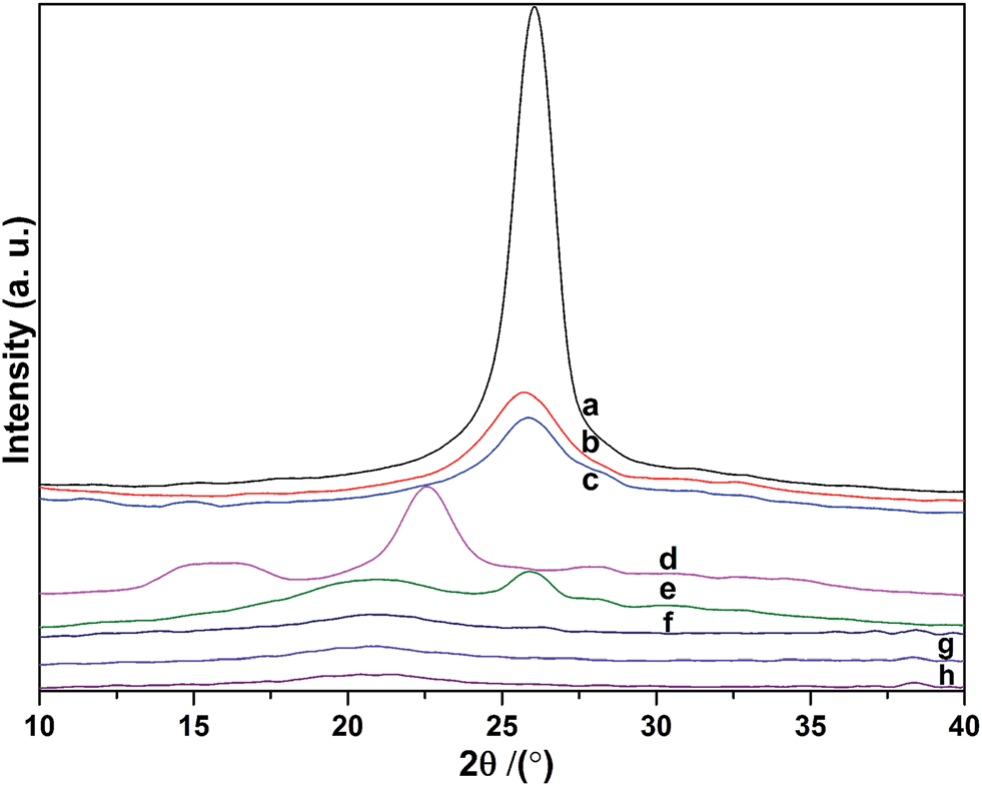
XRD analysis of MWCNTs (a), MWCNT–COOH (b), MWCNT–COCl (c), cellulose (d), MWCNT-cellulose (e), cellulose fibers (f), MWCNT/cellulose fibers (10 wt% MWCNTs) (g), and MWCNT-cellulose/cellulose fibers (10 wt% MWCNTs) (h).

XRD studies of the MWCNTs, MWCNT–COOH, MWCNT–COCl, cellulose, MWCNT-cellulose, cellulose fibers, MWCNT/cellulose fibers and MWCNT-cellulose/cellulose fibers were done to investigate the crystalline behaviors of the samples (Fig. 4). The MWCNT X-ray diffraction pattern shows a 2*θ* diffraction peak angle of about 26°, which is characteristic of the (200) plane reflection of a graphite structure. This sharp peak indicates the neat arrangement of C atoms in the MWCNTs. After carboxylation and acylation (Fig. 4b and c), the full width at half maximum (FWHM) of the sharp characteristic diffraction peak from the MWCNTs was bigger. This indicates that the crystallite sizes were smaller. This could be triggered by some neat structure in the MWCNTs being broken in the processes of carboxylation and acylation. In the spectrum of cellulose (Fig. 4d), the peaks at 16.4° from (101) plane reflection and 22.5° from (002) plane reflection were caused by the transverse arrangement of the crystallites in cellulose I.^24^Fig. 4e–h show X-ray diffraction patterns of MWCNT-cellulose, cellulose fibers, MWCNT/cellulose fibers and MWCNT-cellulose/cellulose fibers. All samples showed the typical cellulose II crystalline form.^25^ This indicated that the cellulose could dissolve completely in AmimCl. In the spectrum of MWCNT-cellulose (Fig. 4d), the peaks at 21.4° and 26° were from the cellulose II crystalline form and graphite structure of MWCNTs, respectively. In addition, as shown in the spectra of the cellulose fibers, MWCNT/cellulose fibers and MWCNT-cellulose/cellulose fibers, the crystallinity of the cellulose fibers was higher than that of the MWCNT/cellulose fibers, and the crystallinity of the MWCNT/cellulose fibers was higher than that of the MWCNT-cellulose/cellulose fibers. This was caused by MWCNTs hindering the formation of crystalline regions among the cellulose chains, leading to a decrease in the degree of crystallinity.^26^ The MWCNTs in the MWCNT-cellulose/cellulose fibers were more uniform than in the MWCNT/cellulose fibers.

**Fig. 5:**
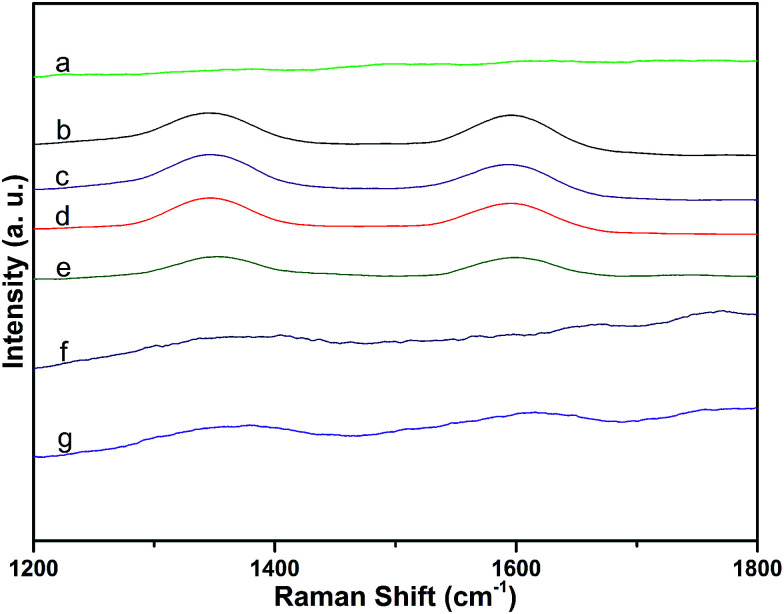
Raman spectra of cellulose (a), MWCNTs (b), MWCNT–COOH (c), MWCNT–COCl (d), MWCNT-cellulose (e), MWCNT/cellulose fibers (10 wt% MWCNTs) (f), and MWCNT-cellulose/cellulose fibers (10 wt% MWCNTs) (g).

Raman spectra of MWCNTs over the range of 1200–1800 cm^−1^ were dominated by two peaks: the D-band (at 1337 cm^−1^, attributed to disorder induced by defects and curvature in the nanotube lattice) and the G-band (at 1577 cm^−1^, due to the in-plane vibration of C–C bonds).^27^ The integral area ratio of the D-band to the G-band (*I*_D_/*I*_G_) can be used to evaluate the extent of any carbon-containing defects.^28^ Raman spectra of the samples are shown in Fig. 5. Compared with *I*_D_/*I*_G_ of MWCNTs (1.1), *I*_D_/*I*_G_ of MWCNT–COOH was higher (1.4). This indicates that the MWCNTs were destroyed during the carboxylation procedure. However, there was no obvious change in the values of MWCNT–COCl and MWCNT-cellulose, compared with MWCNT–COOH. This demonstrated that during the acylation procedure the MWCNTs were not obviously destroyed. The intensities of *I*_D_ and *I*_G_ from MWCNT-cellulose were lower; this could be caused by the MWCNTs being pressured by “coating layer” cellulose, which limited particle vibration.^29^ Due to the MWCNT content in MWCNT/cellulose fibers and MWCNT-cellulose/cellulose fibers being low, the D-band and G-band were weaker. However, the D-band and G-band from the MWCNT-cellulose/cellulose fibers were stronger than from the MWCNT/cellulose fibers; this could be caused by the uniform MWCNTs in the MWCNT-cellulose/cellulose fibers.

### 3.2 Morphology and structure of MWCNT/cellulose fibers and MWCNT-cellulose/cellulose fibers

**Fig. 6:**
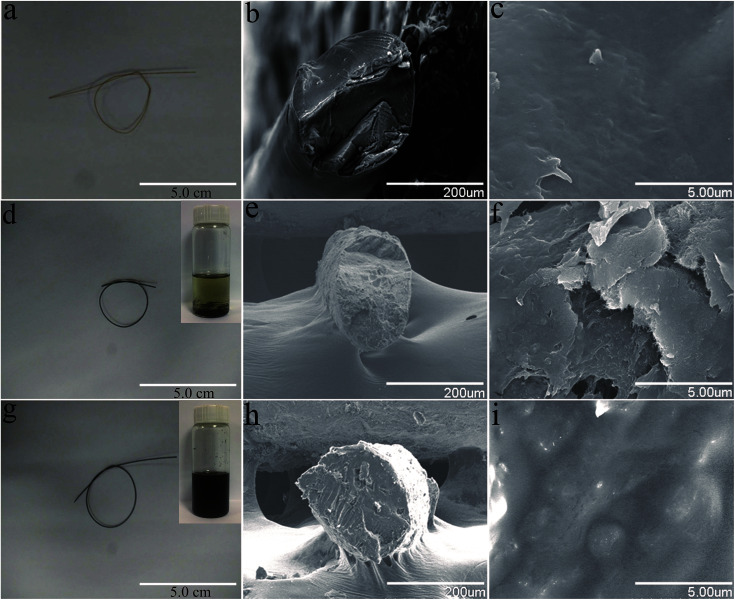
(a, d and g) Photographs of a cellulose fiber, MWCNT/cellulose fiber (10 wt% MWCNTs), and MWCNT-cellulose/cellulose composite fiber (10 wt% MWCNTs). (b, c) SEM images of a cross-sectional fracture of the cellulose fiber. (e, f) SEM images of a cross-sectional fracture of the MWCNT/cellulose fiber (10 wt% MWCNTs). (h, i) SEM images of a cross-sectional fracture of the MWCNT-cellulose/cellulose composite fiber (10 wt% MWCNTs). The inset images in (d) and (g) show MWCNTs and MWCNT-cellulose in AmimCl after 30 days of storage. The concentration of MWCNTs is 0.125 mg ml^−1^.

Fig. 6 shows photographs and SEM images of the cellulose fibers, MWCNT/cellulose fibers with 10 wt% MWCNTs, and MWCNT-cellulose/cellulose composite fibers (containing 10 wt% MWCNTs). The cellulose fibers were nearly transparent. The cross sectional surface of the pure cellulose fibers was flat and featureless in general, illustrating that the resulting cellulose fibers were brittle and the cracks propagate in a planar fashion. However, for the MWCNT/cellulose fibers, the cross sectional surface was extremely rough, which was rather clearly distinctive, compared to the flat fracture surface of the pure cellulose fibers. This indicated that the MWCNTs played a significant role in improving the mechanical properties of the cellulose fibers. Some irregular agglomerated MWCNTs in the cellulose matrix were observed subsequently. The mechanical and electrical properties of the composite fibers would be slightly hampered owing to this aggregation. Remarkably, there were almost no agglomerated particles in the cross sectional surface of the MWCNT-cellulose/cellulose fibers. A homogenous dispersion of MWCNT particles was observed predominantly, compared with the agglomerated particles of the MWCNT/cellulose fibers. The images in Fig. 6d and g show the MWCNTs and MWCNT-cellulose in AmimCl after 30 days of storage, respectively. In Fig. 6d, a large quantity of black MWCNT precipitate is observed in the MWCNT dispersion, whereas the MWCNT-cellulose appears uniform without any MWCNT precipitate (Fig. 6g). These results indicate that the stability of a MWCNT-cellulose dispersion was superior to MWCNTs in AmimCl. These results suggest that cellulose was covalently grafted onto the surface of the MWCNTs, facilitating the dispersion of MWCNTs in the cellulose matrix, and the miscibility between MWCNTs and the cellulose matrix was dramatically promoted *via* MWCNTs wrapped with cellulose. The quality of the anisotropic nano-filler dispersion in the polymer matrix was rather modified in its effectiveness in enhancing mechanical and electrical activities.

**Fig. 7:**
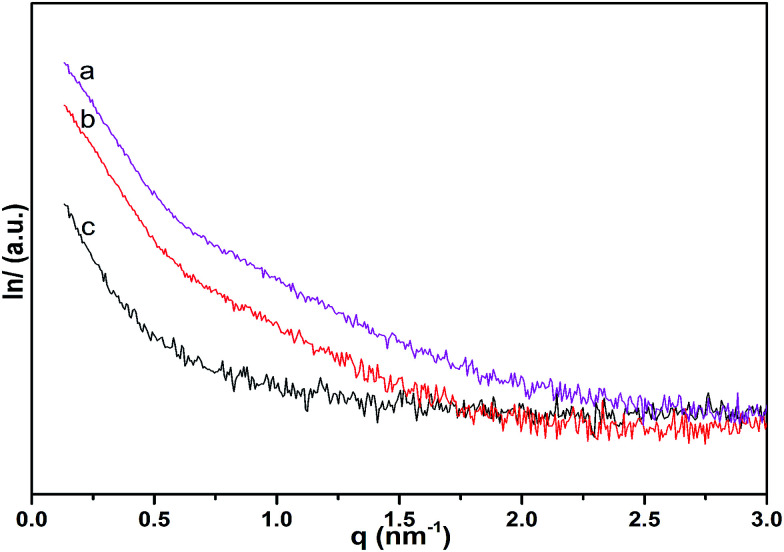
SAXS patterns of MWCNT-cellulose/cellulose fibers (10 wt% MWCNTs) (a), MWCNT/cellulose fibers (10 wt% MWCNTs) (b), and cellulose fibers (c).

SAXS patterns of MWCNT-cellulose/cellulose fibers, MWCNT/cellulose fibers and cellulose fibers are exhibited in Fig. 7. As the MWCNTs join, the intensity of the SAXS diffraction of the fibers increases. However, the SAXS diffraction intensity of MWCNT-cellulose/cellulose fibers is higher than MWCNT/cellulose fibers; this could be caused by irregular agglomerated particles in the MWCNT/cellulose fibers reducing the intensity of the SAXS diffraction, compared with the MWCNT-cellulose/cellulose fibers.

### 3.3 Mechanical properties of MWCNT/cellulose fibers and MWCNT-cellulose/cellulose fibers

**Fig. 8:**
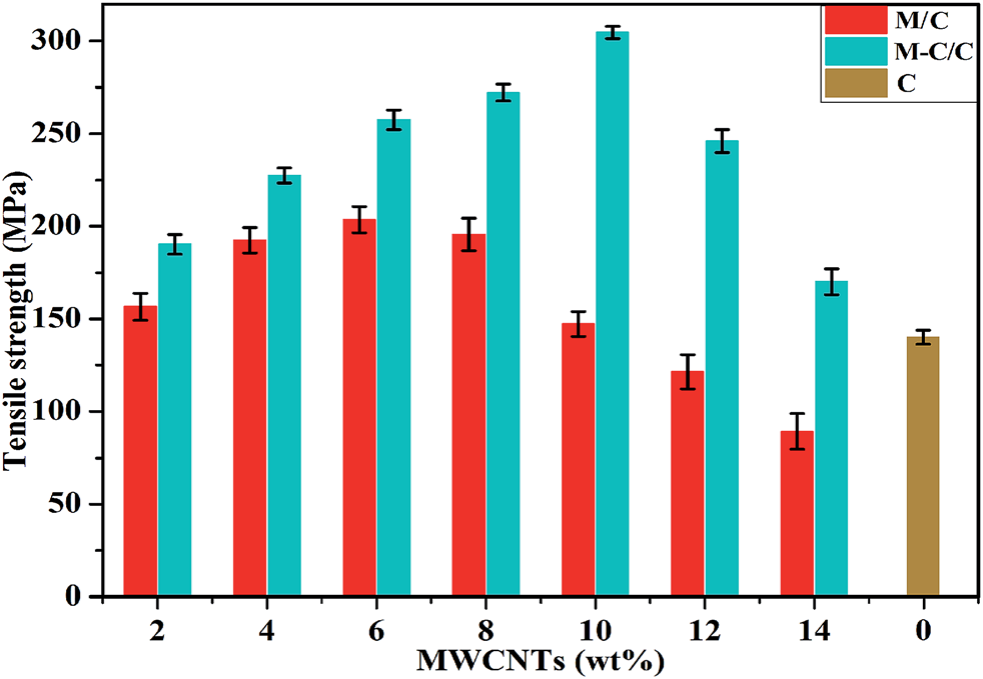
Tensile strengths of MWCNT/cellulose composite fibers (M/C), MWCNT-cellulose/cellulose composite fibers (M-C/C), and cellulose fibers (C).

Furthermore, typical tensile strengths of MWCNT/cellulose fibers, MWCNT-cellulose/cellulose fibers and cellulose fibers were collected respectively, as shown as Fig. 8. The tensile strength of MWCNT/cellulose fibers increased to approximately 203.4 MPa with 6 wt% MWCNTs. However, with MWCNT loading further increasing, the tensile strength decreased inversely, which might be caused by the aggregation of MWCNTs in increasing amounts. For the MWCNT-cellulose/cellulose composite fibers, with an increase in MWCNT-cellulose involved, the tensile strength dramatically improved to 304.6 MPa with 10 wt% MWCNTs, which was almost 106.8% higher than that of MWCNT/cellulose fibers with the same amount of MWCNTs. Meanwhile, this was 117.1% higher than that of cellulose fibers. Moreover, the tensile strength of the MWCNT-cellulose/cellulose fibers was much higher than that of MWCNT/cellulose fibers with the same amount of MWCNTs. The possible reason was that the cellulose covalently grafted along the surface of the MWCNTs facilitated the dispersion of MWCNTs in AmimCl and the cellulose matrix, thus leading to the highly promoted tensile strength performance.

### 3.4 Electrical properties of MWCNT/cellulose fibers and MWCNT-cellulose/cellulose fibers

The electrical conductivity values of the MWCNT/cellulose fibers, MWCNT-cellulose/cellulose fibers and cellulose fibers are shown in Table 1. In terms of MWCNT/cellulose fibers containing 6 wt% MWCNTs, it was demonstrated that the electrical conductivity was up to 8.0 × 10^−3^ S cm^−1^. However, the electrical conductivity values obtained for MWCNT/cellulose fibers containing more than 6 wt% MWCNTs declined, indicating that involving a large amount of MWCNTs could not dramatically increase electrical conductivity, possibly due to aggregation and poor dispersion.^11^ Similar phenomena were observed in a PC/MWCNT fiber composite, as reported by Potschke.^30^ Comparably, the electrical conductivity of MWCNT-cellulose/cellulose fibers improved with the loading of MWCNT-cellulose, up to approximately 1.3 × 10^−1^ S cm^−1^ with 10 wt% MWCNTs, which was almost 108 times higher than that of MWCNT/cellulose fibers with the same amount of MWCNTs. Furthermore, the electrical conductivity of the MWCNT-cellulose/cellulose fibers was higher than that of the MWCNT/cellulose fibers for each amount of MWCNTs. They were dramatically superior to MWCNT/cellulose fibers because of the improved stability from the dispersion of MWCNT-cellulose in AmimCl and the cellulose matrix.

**Table 1:** Electrical conductivity values for MWCNT/cellulose fibers and MWCNT-cellulose/cellulose fibers

	MWCNT-cellulose/cellulose fibers (S cm^−1^)	MWCNT/cellulose fibers (S cm^−1^)
0	0	0
2	4.2 × 10^−4^	1.3 × 10^−5^
4	2.1 × 10^−2^	2.8 × 10^−3^
6	6.8 × 10^−2^	8.0 × 10^−3^
8	8.1 × 10^−2^	4.1 × 10^−3^
10	1.3 × 10^−1^	1.2 × 10^−3^
12	0.9 × 10^−2^	9.3 × 10^−4^
14	7.2 × 10^−3^	6.5 × 10^−5^

## 4. Conclusions

Well-designed MWCNT-cellulose/cellulose fibers with enhanced mechanical and electrical properties were fabricated in AmimCl. XPS spectra of MWCNT–COCl and MWCNT-cellulose were investigated. FTIR data from MWCNT–COCl, MWCNT–COOH, MWCNT-cellulose and cellulose were collected. XRD data from MWCNTs, MWCNT–COOH, MWCNT–COCl, cellulose, MWCNT-cellulose, cellulose fibers, MWCNT/cellulose fibers and MWCNT-cellulose/cellulose fibers were investigated. Raman spectra of cellulose, MWCNTs, MWCNT–COOH, MWCNT–COCl, MWCNT-cellulose, MWCNT/cellulose fibers and MWCNT-cellulose/cellulose fibers were recorded. SAXS patterns of MWCNT-cellulose/cellulose fibers, MWCNT/cellulose fibers and cellulose fibers were investigated. The cross sectional structures of the above composite fibers were investigated using SEM. Cellulose grafting onto the surfaces of the MWCNTs facilitated the dispersion of MWCNTs in the cellulose matrix. The tensile strengths of the obtained MWCNT-cellulose/cellulose composite fibers and MWCNT/cellulose fibers were measured systemically. The tensile strength of the MWCNT/cellulose fiber composite was up to approximately 203.4 MPa with 6 wt% MWCNTs. Comparably, the tensile strength of the MWCNT-cellulose/cellulose fiber composite increased to 304.6 MPa with 10 wt% MWCNTs, which was almost 106.8% higher than that of MWCNT/cellulose fibers with the same amount of MWCNTs. In addition, the electrical performances of MWCNT/cellulose fibers and MWCNT-cellulose/cellulose composite fibers were studied. The highest electrical conductivity of the MWCNT/cellulose fibers was up to 8.0 × 10^−3^ S cm^−1^ with 6 wt% MWCNTs, while the highest electrical conductivity of the MWCNT-cellulose/cellulose composite fibers was promoted to 1.3 × 10^−1^ S cm^−1^ with 10 wt% MWCNTs, which was almost 108 times higher than that of MWCNT/cellulose fibers with the same amount of MWCNTs. Therefore, the well-designed MWCNT-cellulose/cellulose composite fibers could open a new path for applications of wearable electrical devices.

## Conflicts of interest

There are no conflicts to declare.

## Supplementary Material
